# Assessing the Influence of Dyes Physico-Chemical Properties on Incorporation and Release Kinetics in Silk Fibroin Matrices

**DOI:** 10.3390/polym13050798

**Published:** 2021-03-05

**Authors:** Bruno Thorihara Tomoda, Murilo Santos Pacheco, Yasmin Broso Abranches, Juliane Viganó, Fabiana Perrechil, Mariana Agostini De Moraes

**Affiliations:** Department of Chemical Engineering, Institute of Environmental, Chemical and Pharmaceutical Sciences, Universidade Federal de São Paulo, Rua São Nicolau 210, Diadema 09913-030, Brazil; btomoda@gmail.com (B.T.T.); murilopch@outlook.com (M.S.P.); yasbroso@gmail.com (Y.B.A.); julianevigano@gmail.com (J.V.); fabiana.perrechil@unifesp.br (F.P.)

**Keywords:** biopolymer, hydrogel, microparticles, dye release

## Abstract

Silk fibroin (SF) is a promising and versatile biodegradable protein for biomedical applications. This study aimed to develop a prolonged release device by incorporating SF microparticles containing dyes into SF hydrogels. The influence of dyes on incorporation and release kinetics in SF based devices were evaluated regarding their hydrophilicity, molar mass, and cationic/anionic character. Hydrophobic and cationic dyes presented high encapsulation efficiency, probably related to electrostatic and hydrophobic interactions with SF. The addition of SF microparticles in SF hydrogels was an effective method to prolong the release, increasing the release time by 10-fold.

## 1. Introduction

Silk is a natural protein produced in the form of fibers by insect larvae, such as silkworms, spiders, scorpions, mites, and flies, varying in amino acid composition, chemical structure and properties according to its origin [1,2,3]. Two proteins compose the silk obtained from the silkworm cocoons: fibroin and sericin, the former is used for the development of biomaterials and the latter is removed due to reports of adverse events and hypersensitivity [1]. Several studies were carried in the production of silk fibroin (SF) based materials, showing different processes for the incorporation of active compounds, as well as the effects on the delivery system, highlighting the versatility of this protein [2,4,5,6]. SF matrices are mostly hydrophobic and amphoteric ion exchangers, highly dependent on pH, due to the presence of weak acid groups and bases. Thus, SF behaves as an anion exchanger at a pH lower than the isoelectric point (pI = 4.5) and as a cation exchanger at a pH above the pI [7]. SF structure consists of two protein chains, a “heavy” and a “light” chain. The “heavy” chain is mainly formed by ordered hydrophobic macromolecules and “light” chain is formed by polar amino acid residues. Due to these different blocks, SF is capable to perform hydrophilic and hydrophobic interactions, the latter being the main interaction of SF structure [8]. SF has mild processing conditions and can be used to produce hydrogels, microparticles or films [6,9]. 

Hydrogels are three-dimensional and hydrophilic polymeric structures, capable of absorbing large amounts of water or biological fluids, with potential applications in the biomedical area [10]. The physical properties of hydrogels, more than any other class of synthetic biomaterials, are the ones that most resemble living tissues, due to their high water content and their soft, rubbery consistency [11]. SF hydrogels’ formation is a kinetic process that occurs spontaneously from the metastable aqueous solution due to SF molecules’ aggregation tendency, moving from an amorphous conformation (random coil) to a stable conformation of the β-sheet type [12]. Although spontaneous, the formation can be accelerated by adding organic solvents, such as ethanol, which dehydrates SF molecules [12,13]. Moraes et al. [14] developed SF hydrogels capable to sustain diclofenac release for 10 h, showing a high release rate in the first three hours, releasing approximately 80% of the maximum release capacity. Other studies also show a fast release of drugs incorporated into SF hydrogels [14,15], emphasizing the need for strategies to achieve a prolonged and sustained release.

Encapsulation of drugs or bioactive compounds into biopolymeric microparticles is a suitable approach to prolong the release. Besides, as delivery systems, microparticles must protect the therapeutic agent from degradation and denaturation, in addition to controlling the release profile of the compound into the medium [16,17]. SF microparticles are an innovative tool for encapsulating drugs and developing drug delivery systems. Due to the formation of β-sheet structures, SF release devices are formed by crystalline regions, more stable in water, and exhibiting high rates of *in vivo* degradation [18,19]. Thus, aiming to prolong the release profile from SF hydrogels, a combined device of microparticles and hydrogel might have a high potential, allowing to modulate the system to the desired application and to obtain a more prolonged release rate [15,20,21]. 

Numata, Yamazaki, and Naga [15] developed a double release system of fluorescent dyes from SF hydrogels containing SF nanoparticles, aiming at a slow and prolonged release of the dye incorporated in the nanoparticles and a quick release of the dye added in the hydrogel. The release kinetics showed the efficiency of incorporating nanoparticles in the hydrogel, sustaining the release for up to 5 days. However, the influence of the dyes’ properties on the dye-matrix interaction was not evaluated.

In this context, we proposed the synthesis of SF hydrogels containing SF microparticles to release model dyes, aiming at applications as release devices. The novelty of this work is the incorporation of model dyes with different charge, hydrophilicity and molar mass, for mapping the influence of the physico-chemical properties of the compound on the SF matrix and the interaction during incorporation and release, allowing to expand for applications in drugs and other bioactive molecules. The model dyes used were methylene blue (MB), rose bengal (RB), rhodamine B (RhB), and neutral red (NR).

## 2. Materials and Methods

### 2.1. Materials

Silkworm cocoons from *Bombyx mori* were supplied by silk company Bratac (Londrina, Paraná, Brazil). MB, RB, RhB, and NR were purchased from Sigma-Aldrich (St. Louis, MO, USA). All other chemicals were purchased from Synth (Diadema, São Paulo, Brazil). Ultrapure water from the Milli-Q system (Millipore, Burlington, MA, USA) was used.

The nomenclature used to identify the samples was MP for microparticles, HG for hydrogels, SF for silk fibroin, MB for methylene blue, RB for rose bengal, RhB for rhodamine B, and NR for neutral red. The microparticles incorporated with the dyes are identified by ‘MP’ + ‘acronym of the dye or silk fibroin’, and the hydrogels containing the microparticles with the dyes are identified by ‘HG′ + ‘MP’ + ‘acronym of the dye or silk fibroin’.

### 2.2. Preparation of Silk Fibroin Aqueous Solution

Sericin was removed from the silkworm cocoons with 1 g/L aqueous sodium carbonate solution (Na_2_CO_3_) for 30 min in a thermostatic bath at 85 °C. This procedure was repeated three times, and then the SF threads were washed with plenty of distilled water. SF was dried at room temperature for 48 h and cut to an average size of 2 mm to facilitate dissolution. For each 10 g of SF, 100 mL of a ternary solution of calcium chloride, ethanol, and water (CaCl_2_: CH_3_CH_2_OH: H_2_O, 1:2:8 molar) were added and maintained at 85 °C in a thermostatic bath TE-184 (Tecnal, Piracicaba, São Paulo, Brazil) until complete dissolution of SF for a maximum of 90 min.

The SF saline solution (0.1 g/mL) was dialyzed in distilled water for 3 days, at 10 °C, to remove the calcium from the solution and obtain an SF aqueous solution. The dialysis water was changed every 24 h, in the volumetric ratio of 1:15 SF solution:water, with SF aqueous solution concentration of ca. 0.074 g/mL.

### 2.3. Preparation of Silk Fibroin Microparticles

SF microparticles were prepared by the atomization method, in which SF aqueous solution was sprayed into an absolute ethanol coagulation bath. The atomization system consists of a nozzle with a diameter of 0.5 mm (Labmaq, Ribeirão Preto, São Paulo, Brazil), a peristaltic pump TE-BP-01 Mini (Tecnal, Piracicaba, São Paulo, Brazil), and a compressor model OP8.1/30II (Pressure, Maringá, Paraná, Brazil). The SF solution was pumped (flow rate around 0.045 L/h) to the atomizing nozzle, where the solution was broken up into smaller droplets (compressed air pressure at 1 bar). The droplets came into contact with the absolute ethanol under stirring. After forming the microparticles, they were kept at rest for 30 min and then stirred for 10 min.

Each dye solution (1 × 10^−4^ mol/L) was incorporated into the microparticles by adsorption, by immersing 0.1 g of SF microparticles into 10 mL of dye solution and stirring at 30 rpm by a tube revolver (Thermo Scientific, Waltham, MA, USA) until the equilibrium (3 h) at room temperature (24 °C). The dyes used were MB, RB, RhB, and NR. These dyes were selected due to their different properties showed in Table 1. The rational selection was made to verify the influence of the charge, molar mass, and hydrophilicity of the dye in the SF matrices. MB, RB, and RhB are anionic dyes, and NR is a slightly cationic dye at neutral pH. 

### 2.4. Microparticles Characterization

The encapsulation efficiency (EE%) (Equation (1)) was calculated by the ratio between the mass of loaded dye in the microparticles (obtained by mass balance from the residual mass of dye quantified in the supernatant) and the mass of compound added in the fresh dye solution. The quantification of the dyes was performed by spectroscopy in a Genesys 10S UV/Vis spectrophotometer (ThermoScientific, Waltham, MA, USA), using wavelength of 664 nm for MB, 530 nm for RB, 542 nm for RhB, and 520 nm for NR. The calibration curves and fitting parameters are shown in Supplementary Materials, Figure S1.

(1) $$\mathrm{Encapsulation}\;\mathrm{efficiency}\;\left(\%\right)=100\times \;\frac{\mathrm{mass}\;\mathrm{of}\;\mathrm{compound}\;\mathrm{incorporated}}{\mathrm{mass}\;\mathrm{of}\;\mathrm{compound}\;\mathrm{added}}.\;$$

The microparticles’ average size was assessed by optical microscopy using a Primo Star microscope (Zeiss, Oberkochen, Baden-Württemberg, Germany) with a 10× objective. Mean particle diameter and log-normal frequency distribution were calculated from image analysis of at least five (5) different images containing 70–100 microparticles using the ImageJ software. 

The morphology of microparticles was observed in a scanning electron microscope (SEM). The samples were frozen with liquid nitrogen, freeze-dried, and covered with gold in the Sputter Coater, Model K450 (Emitech, Kent, UK), with an Au layer thickness estimated at 200 Å. Samples were observed in a Leo 440i-6070, (LEO Electron Microscopy, Oxford, UK) with an accelerating voltage of 10 kV and a current of 50 mA. 

The microparticles containing the dyes were analyzed with a LEICA DMi8 Confocal Microscope (Leica Microsystems, Wetzlar, Hessen, Germany) with 10× objective at 580 nm emission wavelength, coupled to the LAS X software to observe the dye’s homogeneity through the microparticle matrix.

Fourier transform infrared spectroscopy (FTIR) was performed on SF microparticles before and after the dyes loading to verify possible SF-dye interactions. An Agilent Cary 630 FTIR spectrophotometer (Agilent, Santa Clara, CA, USA) was used in ATR mode, and the samples were analyzed in the wavelength range from 1200 to 2000 cm^−1^, with a resolution of 4 cm^−1^ and 128 scans.

### 2.5. Preparation of Silk Fibroin Hydrogels

The SF aqueous solution (0.074 g/mL) was used to produce the hydrogels and a 50% ethanol solution was slowly added to the SF aqueous solution (10 mL) in a 1:1 ratio [14]. To produce hydrogels with microparticles, 0.1 mg (wet mass) of SF microparticles loaded with dye were added to the SF aqueous solution containing 50% ethanol solution. The mixtures were then added to molds (diameter 4 cm). The hydrogel formation time was measured just after the ethanol addition into the SF solution until the hydrogel’ full gelation, where there is no movement of the liquid solution verified by the tube inversion method. The tube inversion method consists in turning the mold downwards to note whether solution stops to flow due to its own weight.

### 2.6. Hydrogel Characterization

The hydrogel was characterized by a scanning electron microscope (SEM) to observe the morphology. The samples were prepared by supercritical fluid drying [23] to maintain their original structure, fractured in liquid nitrogen, covered with gold and then were observed as previously described in Section 2.4. 

Fourier transform infrared spectroscopy (FTIR) was performed on SF hydrogels with and without microparticles loaded with dyes to verify possible structural changes and SF-dye interactions. The same methodology described at Section 2.4 was used. 

The rheological behavior was evaluated by dynamic oscillatory tests on an MCR 92 rheometer (Anton Paar, Graz, Austria) equipped with a parallel plate geometry with a diameter of 49.96 mm and a gap of 1 mm. For the analysis, the SF solution was mixed with the 50% (*v/v*) ethanol solution, with and without the addition of the microparticles, and immediately transferred to the rheometer plate preheated at 37 °C. A time sweep up to 150 min was carried out, with temperature, strain, and frequency fixed at 37 °C, 1%, and 0.1 Hz, respectively. After 150 min, the gelled samples were subjected to a frequency sweep from 0.01 Hz to 100 Hz at 37 °C and fixed deformation at 1%.

Thermogravimetric analysis (TGA) was performed to verify the behavior, thermostability, and hydrogel degradation peaks under temperature variation. A TGA/differential scanning calorimetry (DSC)1 equipment, coupled with an MX5 microanalytical balance (Mettler Toledo, Columbus, OH, USA) was used. The analysis was carried out in the temperature range of 25 to 600 °C, at a heating rate of 10 °C/min and nitrogen flow rate of 50 mL/min.

The heat flow related to the chemical transitions in hydrogels and microparticles due to temperature changes was obtained from differential scanning calorimetry (DSC) analysis. A Shimadzu DSC-50 (Shimadzu, Nakagyo-ku, Kyoto, Japan) was used with a nitrogen flow rate of 50 mL/min and with a temperature range from 25 to 250 °C, at a heating rate of 10 °C/min.

### 2.7. Release Kinetics of the Dyes

The release kinetics study of SF hydrogels and microparticles loaded with dyes was carried out at 37 °C under constant stirring for up to 24 h in a phosphate-buffered saline solution (PBS) release medium. At certain time intervals up to the equilibrium, a release medium aliquot was collected and analyzed by UV/Vis spectroscopy Genesys 10S (ThermoScientific, Waltham, MA, USA). The medium’s partial renewal was performed and consisted of replacing the sampled aliquot by the same volume of fresh release medium.

In the release of the loaded dyes from the SF hydrogels, it was necessary to add Protease XIV at ca. 300 μL/mL to the release medium [15,19], since the compounds did not release in preliminary tests, persisting the retention in microparticles and hydrogels for at least 72 h. Protease XIV works by degrading the crystalline structure β-sheet of SF hydrogels in smaller fibroin filaments of smaller β-sheet and random coil structures. These smaller structures are again fragmented until reaching a simple SF molecule [19]. For the release from the microparticles, the release medium was only the phosphate-buffered saline solution. 

The mathematical models of Higuchi [24], Peppas [25], Peppas–Sahlin [26], Burst release [27], and Hopfenberg [28] were used to fit the release kinetics data. The fitting parameters were used to determine the dye release mechanism from the SF matrix with Origin software (Originlab). 

## 3. Results and Discussion

### 3.1. Silk Fibroin Microparticles

The dyes were incorporated at neutral pH and, depending on each pKa (Table 1), the dyes showed different charges. MB, RB, and RhB have an anionic character (negative charge), and the NR has a weak cationic character (slightly positive charge) at neutral pH. Besides, MB and RB are hydrophilic dyes but with very different molar mass, while RhB and NR are hydrophobic dyes. Thus, we proceeded with the comparison between RhB (anionic) and NR (cationic) to assess the influence of the charge; MB (319.85 g/mol) and RB (973.67 g/mol) to assess the effect of molar mass; and MB (hydrophilic) and RhB (hydrophobic) to assess the hydrophilicity influence.

**Figure 1:**
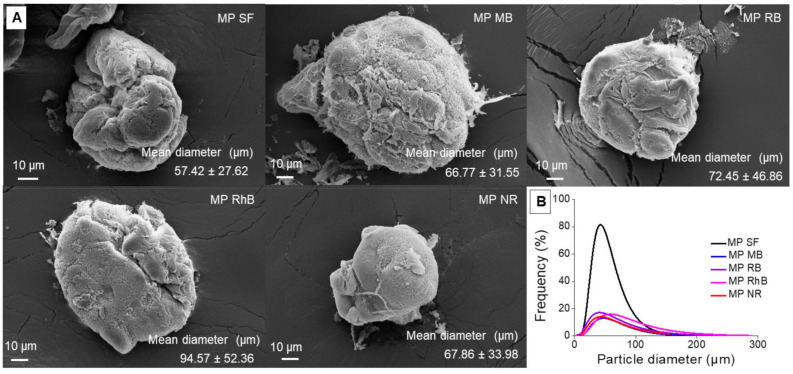
(**A**) Scanning electron microscopy micrograph of silk fibroin (SF) microparticle produced by the atomization method with and without dye loading. The inset values show the mean particle diameter. (**B**) Log-Normal of particle size distribution of SF microparticles.

SF microparticles prepared by the atomization method presented spherical shape (Figure 1A, Figure S2), and no visible change in morphology was observed regarding the dye incorporation into SF microparticle. The microparticle size was controlled by the opening of the atomizer nozzle, liquid and compressed air flow rates [29]. The particle size plays an important role in the release control and the performance of microparticles during drug administration. Particle size generally ranges from 1 to 1000 μm and, together with the structure, has to be defined depending on the desired application [30]. The diameter of SF microparticles prepared in our study was comparable to those prepared by different methods, such as emulsification-diffusion [31] and spray-drying [32]. Nevertheless, it was bigger than those from the coacervation method [33]. The results show an increase of 15% to 65% in the SF microparticles mean diameter when the dyes were incorporated. However, the Tukey-Kramer test analysis at 95% confidence indicates no significant difference between the mean diameters of the loaded microparticles regarding the raw SF microparticle. Figure 1B presents the Log-normal particle size distribution of SF microparticles, which confirmed the homogeneity of microparticles. In addition to the increase of particle size, the encapsulation of dyes also led to an increase in the polidispersity of the samples.

**Figure 2:**
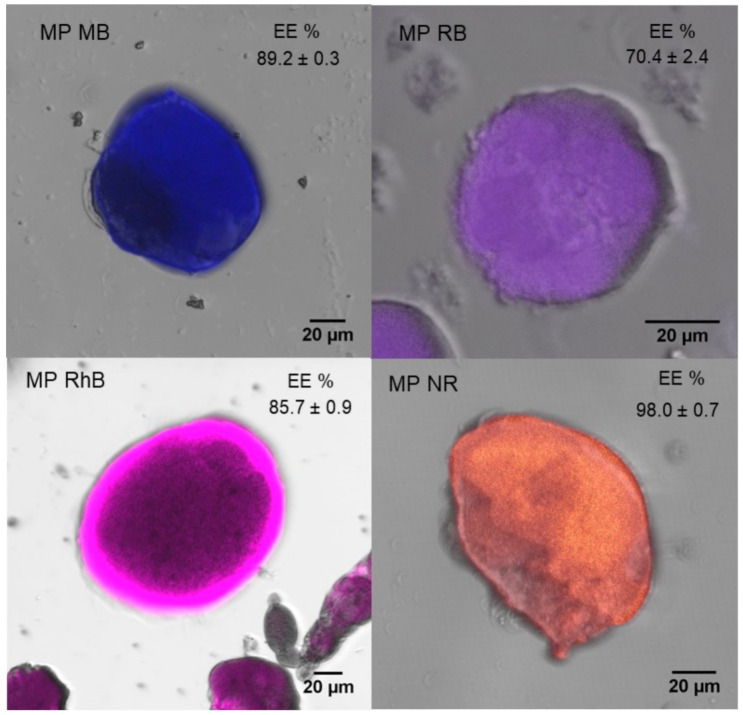
Confocal microscopy images of SF microparticles produced by the atomization method and loaded with dyes. The inset values represent the encapsulation efficiency of the dyes.

From the confocal micrographs (Figure 2), it is possible to infer that the dyes were incorporated and evenly dispersed in the SF microparticles, since the fluorescence associated with the dyes is uniform and without flaws in the microparticles (Figure S3). 

The dye encapsulation efficiency on SF microparticles is presented in Figure 2. The NR, which has a positive net charge, showed the highest encapsulation efficiency, due to an electrostatic interaction between the dye and SF, which has a negative net charge after dialysis. Besides, the high encapsulation efficiency of NR can be related to the capacity of SF to make hydrophobic interactions, having great interaction with this compound. In contrast, RB has a hydrophilic and anionic character, having less interaction with SF and, consequently, less adsorption capacity in SF microparticles. Although MB has a hydrophilic and anionic character, the MB molar mass is smaller than the RB, which may have influenced the incorporation due to a greater barrier to adsorption on the SF microparticle surface. Moreover, the results in Figure 2 reveal that all dyes have high encapsulation efficiency (>70%) in the SF microparticles, even for the RB dye.

Numata, Yamazaki, and Naga [15] showed an encapsulation efficiency of 35% for the texas red dye and 55% for the RhB and fluorescein isothiocyanate in the SF nanoparticles for particle diameter ranging from 175 to 209 nm. The results presented by these authors can be justified by the different dyes characteristics and the incorporation methodology, in which the dye was added to the fibroin solution before the preparation of the microparticles. The texas red is a hydrophilic and anionic dye, with fewer interactions and less incorporation in the microparticles. The RhB, on the other hand, is a hydrophobic and cationic dye and the fluorescein isothiocyanate is a hydrophobic and anionic dye. Therefore, due to hydrophobic interactions, it was expected that RhB and fluorescein isothiocyanate could be incorporated in greater quantities in the SF nanoparticles. Additionally, fluorescein isothiocyanate has a greater hydrophobic character, presenting a greater microparticle and dye interaction (mainly governed by hydrophobic interactions), and a dye release for up to 24 h [15].

**Figure 3:**
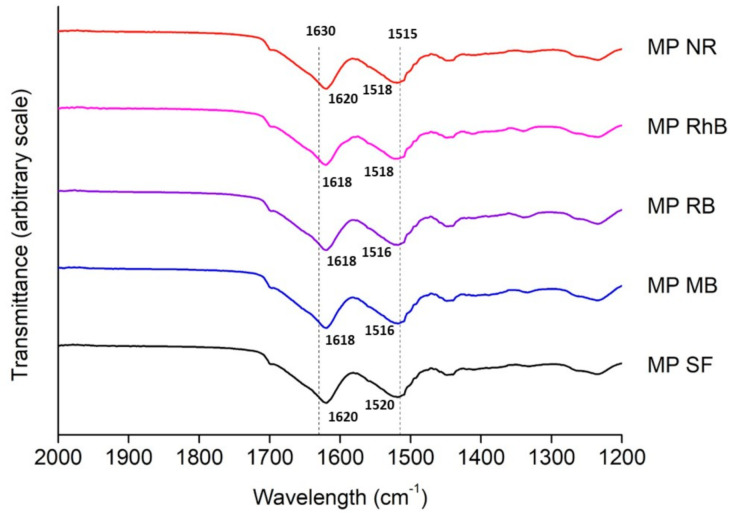
Fourier transform infrared spectroscopy (FTIR) spectra of SF microparticles with and without dyes.

The FTIR spectra of the SF microparticles with and without dyes are presented in Figure 3. The amide I bands are located close to 1620 cm^−1^, and the amide II bands are located between 1516 and 1520 cm^−1^. The stable β-sheet structure (silk II) is mostly found when the wavelengths of amide I, II, and III are presented in 1630, 1515, and 1260 cm^−1^, respectively, while the α-helix (silk I) is found in 1660, 1540, and 1230 cm^−1^ [34]. Thus, it is possible to confirm that the SF microparticles with and without the model dyes are in the most stable conformation, i.e., β-sheet structure. This result was expected for SF microparticles because the microparticles were produced by the atomization method, where the SF solution was atomized in the coagulation bath of ethanol, an organic solvent that induces β-sheet formation in SF [35]. The same trend was observed for the SF microparticles loaded with dyes, since the microparticles were produced and stabilized in ethanol before the incorporation of the dyes.

### 3.2. Silk Fibroin Hydrogels

**Figure 4:**
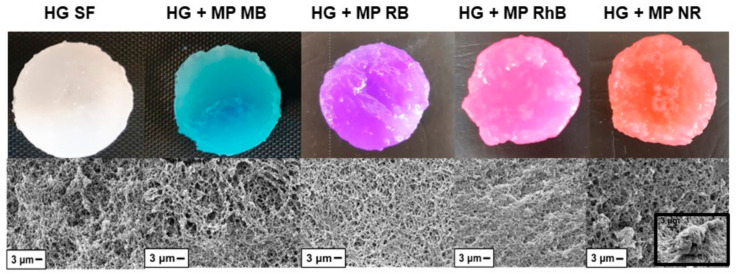
Photographs and scanning electron microscope (SEM) fracture micrographs of SF hydrogels with and without microparticles loaded with dye.

Figure 4 presents the photographs of the SF hydrogels (4 cm diameter). The microparticles were dispersed in the hydrogel, which acquired the color of the dye (Figure 4). Moraes et al. [14] prepared SF hydrogels using the same methodology (mixing with ethanol) in approximately 27 min, which is in accordance with the gelation time of SF hydrogel in the present study. Matsumoto et al. [36] verified that the gelation process of SF depends on several variables, such as protein concentration, temperature, and pH. Kim et al. [37] achieved shorter gelation time and better mechanical properties of SF hydrogels by increasing SF concentration. In this study, we chose to keep SF concentration constant and decrease of gelation time was achieved by addition of ethanol. In addition, it was observed that the incorporation of loaded microparticles increased the gelation time of the SF hydrogels. The gelation time of SF with the loaded microparticles estimated by the tube inversion method ranged from 80 to 85 min, except for the HG + MP NR, in which gelation time was 110 min.

The SEM micrographs (Figure 4) indicate that all SF hydrogels (with or without microparticles) have an interconnected porous structure forming the hydrogel matrix. Figure 4 (HG + MP NR inset) shows a micrograph emphasizing the presence of microparticles in the hydrogel network, indicating the effectiveness of incorporating microparticles into hydrogels.

**Figure 5:**
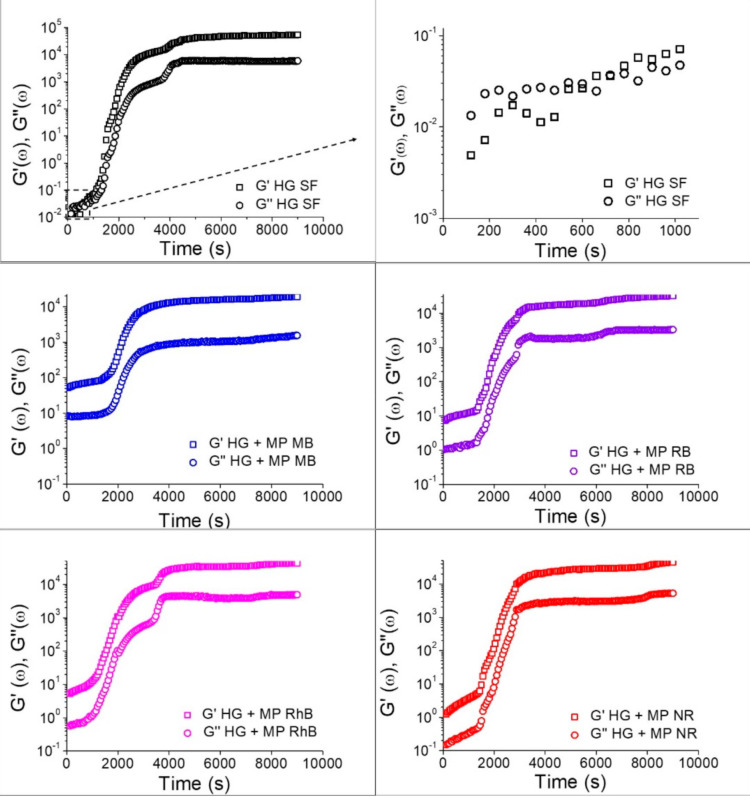
Storage modulus (G′) and Loss modulus (G″) as a function of time of the SF hydrogel and SF hydrogels containing the microparticles loaded with dyes.

In order to understand the effect of time on the formation of hydrogels, rheological tests were performed with time sweep measurements, to verify the behavior of the SF hydrogel through the observation of the storage (G′) and loss moduli (G″) at a constant frequency (Figure 5). For the hydrogels without microparticles, at the beginning of the gelation process, G″ is greater than G′, in which both G″ and G′ are increasing linearly, and, at around 600 s, there is a crossover point and G′ becomes higher than G″, increasing the viscoelasticity due to the gelation process. The linear increase in G′ after the crossover point of HG SF in Figure 5 indicates an increase in viscoelasticity that occurs due to the formation of β-sheet structures [38]. Hydrogels containing the microparticles have G′ greater than G″ from the beginning of the process, but there was a delay in the curve, indicating a longer time required to start the gelation, ranging from 1500 to 2000 s. Besides, at the end of the gelation process, there is a plateau in which the G′ value for hydrogels without the addition of microparticles was about 10-fold greater than G′ for hydrogels containing microparticles. These results indicate that the addition of microparticles containing dyes reduced the interactions between the SF molecules, taking a long time to start the hydrogel formation and resulting in a gel with less elastic character. The comparison between samples containing microparticles with different dyes showed that the dye type had little influence on gelation time and viscoelastic properties. These results are in agreement with the gelation time observed by the tube inversion method.

**Figure 6:**
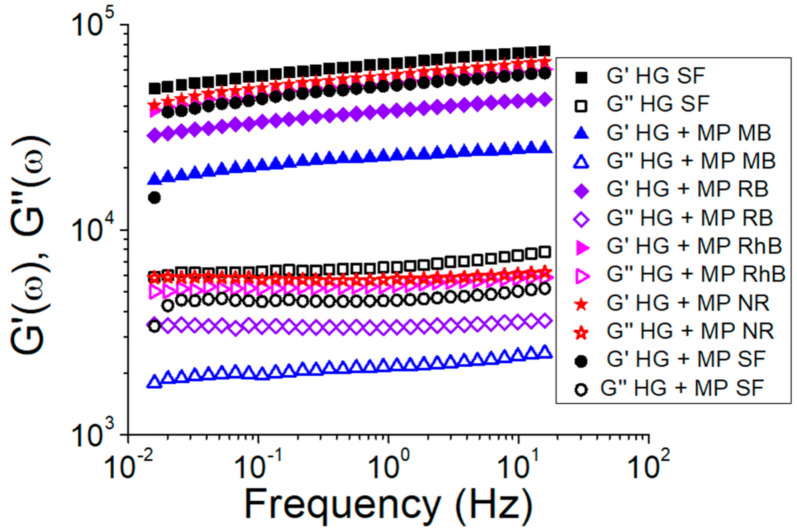
Storage (G′) and loss moduli (G″) as a function of the frequency of SF hydrogels with and without microparticles loaded with dyes. Closed points refer to the storage modulus (G′) and open points refer to the loss modulus (G″).

Frequency sweep measurements were also performed to evaluate the effect of the microparticles incorporation on the SF hydrogels’ structure (Figure 6). All samples showed G′ higher than G″ and both moduli practically independent on frequency, which is a mechanical spectrum characteristic of gel [39]. Figure 6 also shows that both G′ and G″ decreased due to the microparticles addition in the hydrogel. Thus, the reduction of G′ and G″ indicates that the SF hydrogels structure was affected by the presence of the microparticles. Nejadnik et al. [40] found the same influence due to incorporating calcium phosphate nanoparticles in hyaluronic acid hydrogels. The storage modulus (G′) is directly proportional to the gel behavior [41], indicating that the SF hydrogel without microparticles showed a greater gel behavior. From Figure 6 and the gelation time results (Figure 5), it appears that the hydrogels containing microparticles with the largest gelation times also showed a greater G′, indicating hydrogels with a more structured gel network. These results can be related to the hydrogel microstructure (Figure 4), since the HG + MP RhB and HG + MP NR, which showed a more compact structure by SEM, also exhibit a more structured gel network by rheological tests.

**Figure 7:**
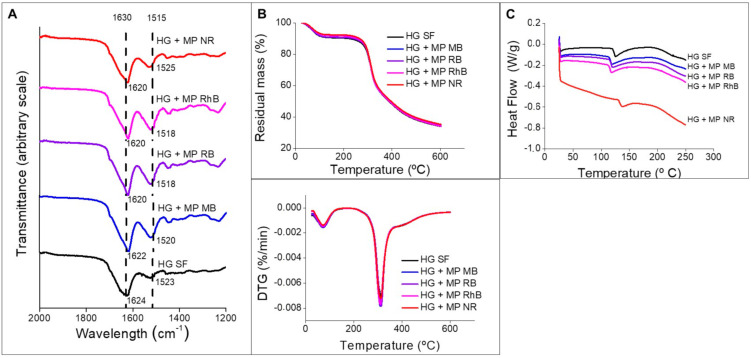
(**A**) FTIR spectra of SF hydrogels with and without microparticles loaded with dyes. (**B**) Thermogravimetric analysis (TGA) and derivative thermogravimetry (DTG) thermograms of SF hydrogels with and without microparticles loaded with dyes. (**C**) DSC thermogram of SF hydrogels with and without microparticles loaded with dyes.

Figure 7A shows the FTIR spectra of the SF hydrogels with and without the microparticles loaded with the dyes. The amide I bands are located around 1620 to 1637 cm^−1^ and the amide II bands are located between 1523 and 1525 cm^−1^. The SF hydrogels with and without the microparticles containing the dyes show β-sheet conformation, similar to the SF microparticles (Figure 3). just like in microparticles, β-sheet conformation was expected in hydrogels due to the gelation and aggregation of SF molecules. Comparing the SF hydrogel’s spectra with the hydrogels containing the microparticles, it was observed that there was no chemical change in the hydrogel structure by incorporating the microparticles. Additionally, new bands related to the dyes could not be observed, which is expected because the point analysis method (FTIR-ATR) was used. Therefore, peaks related to the dyes would only be observed if the FTIR diamond tip was placed exactly on top of a microparticle containing the dye.

Regarding TGA (Figure 7B), a peak of degradation around 100 °C and another peak of degradation around 300 °C were observed. The first peak is related to the water loss from the hydrogel, while the second peak is related to the side chains of the residual amino acid groups [42], which is in accordance with the literature [12]. The degradation temperature did not change when microparticles were incorporated into the SF hydrogel. Thus, there is no significant change in the hydrogel’s thermal stability due to the microparticles’ presence.

The DSC thermogram (Figure 7C) indicates a peak between 118 °C for the SF hydrogel, at 115 °C for the hydrogel containing microparticles with MB, RB, and RhB, and at 130 °C for NR, probably related to SF glass transition temperature [43]. The rise in temperature at this peak indicates that there is a strong interaction between SF and NR. This interaction is related to the SF microparticles’ capacity to adsorb the NR dye in large quantities (Figure 2), due to the dye hydrophobic and cationic character. 

### 3.3. Release Kinetics of the Dyes

**Figure 8:**
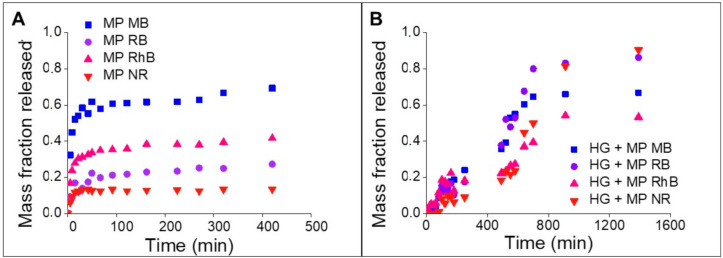
Release kinetics. (**A**) SF microparticles loaded with dyes. (**B**) SF hydrogels containing the microparticles loaded with dyes.

The dyes release from SF microparticles exhibited a rapid release profile (Figure 8A), reaching equilibrium in approximately 90 min. MB had the highest mass fraction released, followed by RhB, RB, and NR. The dye with a hydrophobic character and positive charge, i.e., NR, showed a lower mass fraction released, due to the strong electrostatic and hydrophobic interactions with fibroin. For the MB dye, a higher release into the medium was already expected due to the dye’s anionic and hydrophilic characteristics, which indicates a weak interaction with SF. RB is an anionic and hydrophilic dye, just like the MB dye; however, the lower release of this dye is probably influenced by the RB molecule’s size, which has a molar mass three times greater than MB, which can hinder the diffusion and release of the dye in the release medium.

Table 2 shows the models’ parameters obtained by the mathematical fitting. All models presented a satisfactory fit (R² > 0.90) to the experimental data. The parameter *‘n’* of the Peppas and Peppas–Sahlin models is equal to 0.45, indicating that Fickian Diffusion for spherical geometry is the predominant mechanism in releasing dyes from SF microparticles [25,44].

The release profile of the SF hydrogels containing the microparticles loaded with dyes showed a slow and prolonged release, persisting for approximately 900 min (15 h) (Figure 8B). Numata, Yamazaki, and Naga [15] observed the release of approximately 90% of RhB incorporated in SF hydrogels within 1 h. On the other hand, FITC, the other model drug used in the study, was loaded on SF nanoparticles incorporated in the hydrogels and was totally released just after 5 days.

Comparing the time to release the dyes from the loaded microparticles and SF hydrogel containing the loaded microparticles, there is a 10-fold increase in the release time, showing that the strategy of incorporating the microparticles in the hydrogel is effective in reducing the dyes’ release time. 

For the release in the hydrogels, it was necessary to add Protease XIV, also used by Numata, Cebe, and Kaplan [15,19] since the dyes did not release in preliminary tests, in which the retention of dyes in the SF hydrogels containing the microparticles persisted for at least 72 h. The addition of Protease XIV was not necessary for dyes release from SF microparticles, since dyes were incorporated on SF microparticles by adsorption, being retained on SF microparticles surface, which allowed dyes release to the medium more easily. The different behaviors on dye release from SF microparticles and SF hydrogels are probably related to the dye availability to the medium: in the microparticles, the dyes are adsorbed to the surface, while in the hydrogels the dyes are located inside the SF hydrogel structured network, retained in the microparticles surface. Protease XIV was employed to degrade the stable structure crystalline β-sheet of SF hydrogels, leading the SF filaments to break into smaller β-sheet and random coil structures.

The release of RB dye showed different behaviors: a small dye fraction was released from the SF microparticles loaded with dyes and a high fraction from the SF hydrogel containing the microparticles. This behavior can be related to the fact that RB has a bigger molar mass than the other dyes, influencing its diffusion from the microparticles surface to the release medium. On the other hand, the addition of Protease XIV in the hydrogel release assays led to a higher release of RB, since restriction of RB diffusion was broken up by the smaller SF structures.

Table 3 shows the parameters obtained by fitting the Peppas, Peppas–Sahlin, Hopfenberg, and Higuchi models to the experimental data of dyes released from the SF hydrogels containing microparticles loaded with dyes. MB had the best fitting (0.85 < R² < 0.96), and the Peppas model was the one that best described the behavior of the dyes’ release from SF hydrogel matrices (R² > 0.84). The Peppas–Sahlin, Hopfenberg, and Higuchi models did not fit well the release data; thus, they were not adequate to describe the release behavior of all dyes.

Considering only the Peppas model, the parameter *‘n’* indicates that the Case-II Transport mechanism [25] is associated with a release dependence on matrix degradation. This behavior was predominant in the release of the RB and NR. The anomalous transport mechanism was predominant in the MB and RhB release, having a greater influence of the Fickian Diffusion mechanism in the RhB release and a greater influence of the Case-II Transport mechanism in the MB release. As the releases of the dyes from SF hydrogels incorporated with loaded SF microparticles are controlled mainly by the Anomalous Transport and Case-II mechanisms, there is little influence of the Fickian Diffusion mechanism [25]. The influence of the Case-II Transport mechanism, that is, the release dependence on matrix degradation, can be noticed in all the release curves since the fraction of released compound presents a lag time behavior and slower release behavior. Such behavior was expected because it was necessary the addition of Protease XIV to induce the SF hydrogel matrices degradation [19]. In this way, the fraction of released dye can remain stable or increase, as the hydrogel matrix is degraded, allowing the dye to leave its network.

The incorporation of the microparticles loaded with dyes in the SF hydrogels allowed a slow and prolonged dyes release, although accelerated by the action of the enzyme Protease XIV. The release of dyes loaded in the microparticles incorporated in the hydrogels proved to be dependent on the degradation of the hydrogel and microparticles by the Protease XIV, which, by degrading the β-sheet structure of fibroin, facilitates the dyes release into the medium.

## 4. Conclusions

SF microparticles and hydrogels were produced aiming the development of a prolonged release device. The SF microparticles showed high adsorption capacity of dyes, ranging from 70 to 98%. Positive charged dye (NR) showed the high adsorption capacity, which was related to the electrostatic interaction with the SF microparticles (negatively charged), in addition to hydrophobic interactions. The MB, RB, and RhB have negative charges under the study conditions (neutral pH), even though they exhibited adsorption capacity of at least 70%, indicating the presence of minor hydrophilic interactions (MB and RB) and hydrophobic interactions (RhB) with the SF microparticles. Thus, although fibroin mostly performs hydrophobic interactions, the influence of hydrophilic and electrostatic interactions was observed in the loading of dyes into SF microparticles. These interactions allow a more effective incorporation of the dyes in the microparticles.

The SEM images showed that incorporating the loaded microparticles in the SF hydrogels was carried out effectively. The SF hydrogels chemical structure was not affected by the addition of the microparticles. The rheological and thermal properties, on the other hand, were affected by the microparticles, probably due to changes in the hydrogel network organization.

The dyes release from the microparticles reached equilibrium in approximately 90 min, while from the hydrogel containing the SF microparticles persisted for approximately 900 min, exhibiting a release 10 times longer. Besides, the dyes release from the SF microparticles showed Fickian Diffusion as the predominant mechanism. In contrast, SF hydrogel containing SF microparticles presented the Anomalous Mechanism and the Transport of Case-II as the predominant mechanisms, indicating that the dye’s release is dependent on the matrix degradation. 

The strategy used to incorporate microparticles in hydrogels was effective in prolonging the release. Moreover, it was possible to better understand the influence of the charge, hydrophilicity, and size of the molecules on the adsorption and the release of dyes from SF matrices. Thus, this study opens new possibilities for the development of prolonged-release devices, and it can also be expanded to applications with other molecules, such as drugs and bioactive compounds.

## Figures and Tables

**Table 1:** Properties of the dyes used to load the SF microparticles.

Dye	Hydrophilicity	Solubility in Water (mg/mL) *	Molar Mass (g/mol) *	pKa **
MB	Hydrophilic	50	319.85	3.14
RB	Hydrophilic	100	973.67	4.7
RhB	Hydrophobic	7.8 [22]	479.02	3.7
NR	Hydrophobic	10	288.78	6.8

* Data obtained from supplier safety datasheet. ** Data obtained from PubChem.

**Table 2:** Parameters obtained by fitting the Peppas, Peppas–Sahlin, Higuchi, and burst release models to the SF microparticles’ dye release data.

Model	Equation	Parameters	MP MB	MP RB	MP RhB	MP NR
Peppas	$\frac{M_{t}}{M_{\infty }}=Kt^{n}$	K (1/s^n^)	0.0238	0.0054	0.0128	0.0050
		n	0.4500	0.4504	0.4500	0.4500
		R²	0.9009	0.9250	0.9799	0.9656
Peppas–Sahlin	$\frac{M_{t}}{M_{\infty }}=K_{1}t^{n}+K_{2}t^{2n}$	K_1_ (1/s^n^)	0.0224	0.0050	0.0129	0.0050
		K_2_ (1/s^2n^)	0.0000	1.48 × 10^−5^	0.0000	0.0000
		n	0.4500	0.4500	0.4500	0.4500
		R²	0.9067	0.9083	0.9688	0.9531
Higuchi	$\frac{M_{t}}{M_{\infty }}=K_{h}\sqrt{t}$	K_h_ (1/s^0.5^)	0.0157	0.0037	0.0091	0.0035
		R²	0.9154	0.9346	0.9739	0.9593
Burst release	$\frac{M_{t}}{M_{\infty }}=Kt^{n}+B$	K (1/s^n^)	0.0206	0.0043	0.0124	0.0048
		n	0.4500	0.4748	0.4500	0.4500
		B	0.0422	0.0055	0.0071	0.0035
		R²	0.9235	0.9076	0.9718	0.9564

**Table 3:** Parameters obtained by fitting the Peppas, Peppas–Sahlin, Hopfenberg, and Higuchi to the release data of the dyes loaded in the microparticles incorporated in the SF hydrogels.

Model	Equation	Parameters	HG + MP MB	HG + MP RB	HG + MP RhB	HG + MP NR
Peppas	$\frac{M_{t}}{M_{\infty }}=Kt^{n}$	K (1/s^n^)	0.002	0.002	0.012	0.001
		n	0.813	0.890	0.483	0.890
		R²	0.960	0.928	0.914	0.844
Peppas–Sahlin	$\frac{M_{t}}{M_{\infty }}=K_{1}t^{n}+K_{2}t^{2n}$	K_1_ (1/s^n^)	0.002	-	-	0.000
		K_2_ (1/s^2n^)	0.001	-	-	3.81 × 10^−4^
		n	0.450	-	-	0.527
		R²	0.949	-	-	0.863
Hopfenberg	$\frac{M_{t}}{M_{\infty }}=1-{\left(1-\frac{k_{0}t}{c_{0}r}\right)}^{n}$	k_0_ (cm²/mol.s)	0.000	0.000	-	-
		R²	0.953	0.943	-	-
Higuchi	$\frac{M_{t}}{M_{\infty }}=K_{h}\sqrt{t}$	K_h_ (1/s^0.5^)	0.013	0.016	0.011	0.011
		R²	0.845	0.768	0.920	0.690

## Data Availability

The data presented in this study are available on request from the corresponding author.

## Supplementary Materials

The following are available online at https://www.mdpi.com/2073-4360/13/5/798/s1. Figure S1: Calibration curve and fitting parameters for MB, RB, RhB and NR dyes, Figure S2: Optical microscopy image obtained from SF microparticles loaded with dyes, Figure S3: Confocal microscopy images of SF microparticles loaded with dyes.
