# Supplementary material for: Assessing the Influence of Dyes Physico-Chemical Properties on Incorporation and Release Kinetics in Silk Fibroin Matrices
Source: Polymers (Basel). 2021 Mar 5;13(5):798. doi: 10.3390/polym13050798 (PMC7961930; doi:10.3390/polym13050798)
Supplement: Supplementary file 1 [file polymers-13-00798-s001.pdf]

polymers

MDPI

Supplementary Materials:

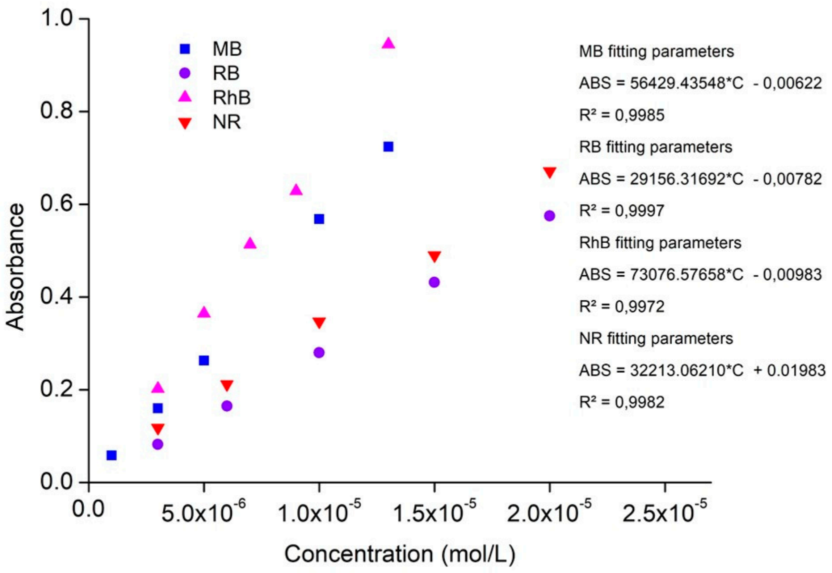

Figure S1. Calibration curve and fitting parameters for MB, RB, RhB and NR dyes.

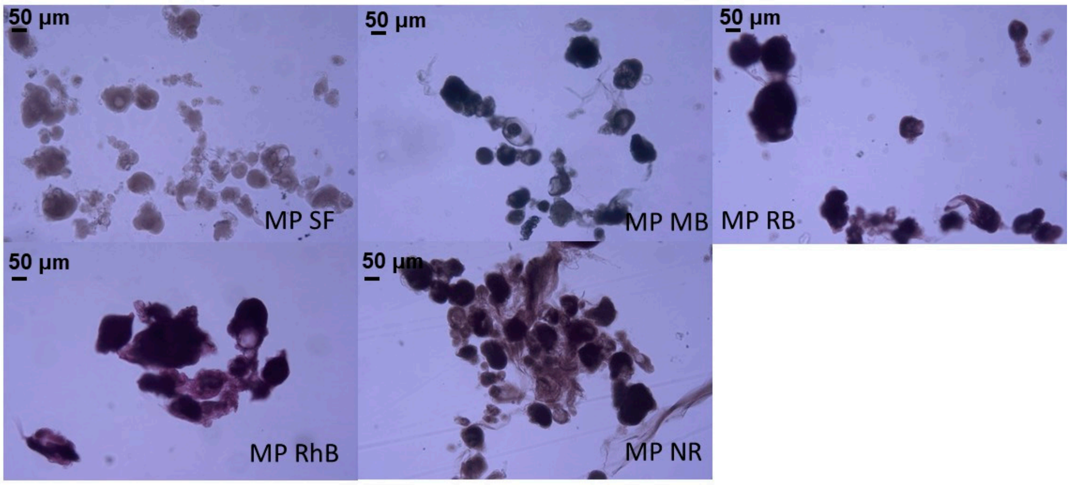

Figure S2. Optical microscopy image obtained from SF microparticles loaded with dyes.

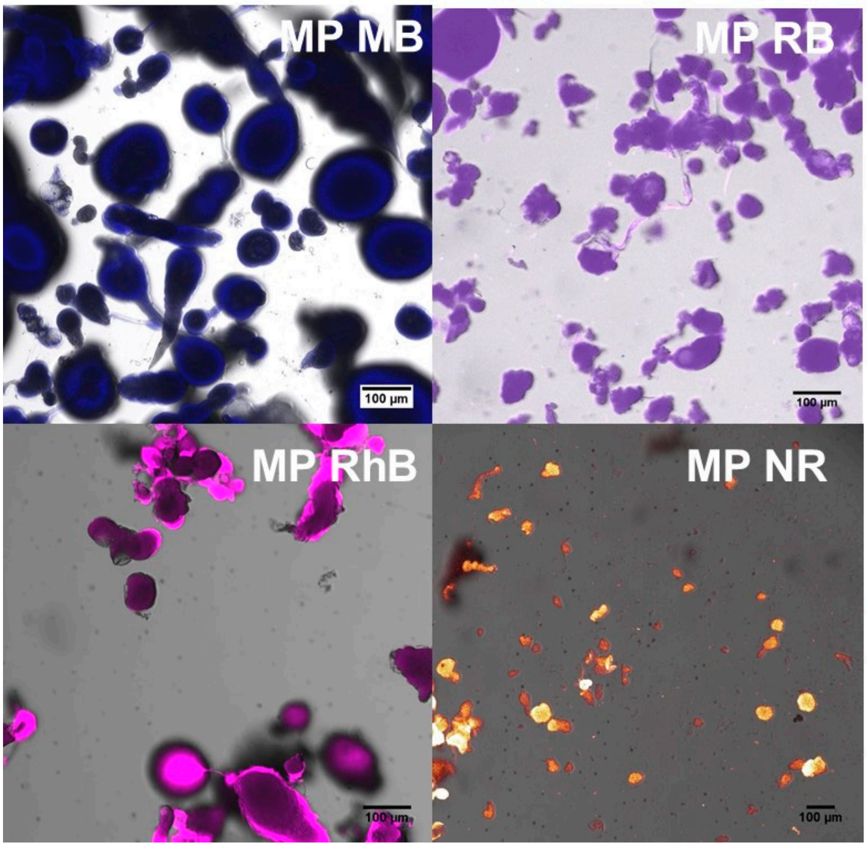

Figure S3. Confocal microscopy images of SF microparticles loaded with dyes.
